# Sjögren's Syndrome: Epidemiology, Classification Criteria, Molecular Pathogenesis, Diagnosis, and Treatment

**DOI:** 10.1002/mco2.70297

**Published:** 2025-07-11

**Authors:** Ying Hu, Bin Wen, Yunfei Zhang, Xiaocui Wang, Xuemei Duan, Haonan Li, Yufeng Fan, Huifeng Shang, Yukai Jing

**Affiliations:** ^1^ Department of Clinical Laboratory, Third Hospital of Shanxi Medical University, Shanxi Bethune Hospital, Shanxi Academy of Medical Sciences Tongji Shanxi Hospital Taiyuan China

**Keywords:** classification criteria, diagnosis, epidemiology, molecular pathogenesis, Sjögren's syndrome, treatment

## Abstract

Sjögren's syndrome (SS) is a chronic autoimmune disorder characterized by T‐cell‐mediated B‐cell hyperactivity and cytokine production, clinically manifesting, dry mouth and eyes, accompanied by pain and fatigue. The disease may progress from asymptomatic glandular involvement to systemic manifestations or even lymphoma. The pathogenesis of SS is intricate, involving a multifaceted interplay of genetic, environmental, and immunological factors. There is still uncertainty regarding the effectiveness of SS‐targeted treatments, due to the significant diversity in disease phenotypes and potentially varying responses to immunomodulatory therapies, stringent enrollment criteria and adoption of outcome metrics in clinical trials may partially explain the failure of many trials to achieve their primary outcomes. Despite the current lack of effective treatments, recent advancements have been made in epidemiology, the development of classification criteria, and the establishment of systems for assessing disease activity. Notably, enhanced insights into the pathogenesis have paved the way for the potential development of targeted therapies. This review aims to systematically synthesize the latest research advancements in the epidemiological characteristics, diagnostic criteria, molecular mechanisms, and clinical manifestations of SS, thereby providing a scientific foundation for the development of future therapeutic strategies.

## Graphical abstract

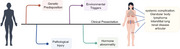

## 1 Introduction

Sjögren's syndrome (SS) is a complex autoimmune disease, characterized by malfunction and destruction of exocrine glands, resulting in classic symptoms of dry eye, dry mouth, fatigue, and joint pain [1]. Historically, SS has been classified into two categories based on clinical manifestations: when symptoms occur independently, it is referred to as primary Sjögren's syndrome (pSS); when symptoms coexist with other systemic autoimmune diseases, it is termed secondary SS. Currently, clinical research primarily focuses on pSS [2]. This disease predominantly affects women, with female‐to‐male ratios reported between 9:1 and 28:1 [3, 4]. The onset typically occurs between 40 and 50 years [3].

The high variability in clinical manifestations of pSS poses a significant challenge in determining the global disease burden of this condition. Over the past three decades, the heterogeneity of pSS has become increasingly evident, reflecting the growing efforts to understand, diagnose, and characterize the disease through clinical and biological research [5, 6]. Despite its heterogeneity, the majority of patients experience similar symptoms, including dryness, pain, and fatigue. Proteomic stratification techniques have recently been employed more often to manage the vast pathophysiological and clinical variability in SS, yielding interesting and promising results. Therefore, a comprehensive and in‐depth understanding and evaluation of pSS remain crucial.

Building on the previous series, this review provides an updated literature overview of the pathogenesis and clinical features of SS. We critically examine methodological considerations concerning the classification standards for pSS and perform an extensive review of its prevalence, incidence, and mortality rates across various regions and time periods. Finally, during the precision medicine period, our attention is on the recent advancements in SS treatment and published clinical trials, seeking to identify possible shortcomings and existing research gaps that might clarify the ineffectiveness of certain systemic therapies.

## 2 The Epidemiology of SS

### 2.1 Prevalence and Sex Bias of SS

Epidemiological studies indicate that current investigations into pSS prevalence are predominantly conducted in Asia and Europe, with relatively fewer studies in the Americas and a notable absence of epidemiological data from Africa. From a methodological perspective, existing research primarily employs two design approaches: the initial approach is a sampling survey technique that determines prevalence rates within randomly selected specific populations through questionnaire screening combined with diagnostic testing; the second is a population‐based approach, aimed at comprehensively assessing the overall prevalence within a specific region or country. Comparative methodological studies suggest that the sampling survey method may carry a risk of overestimating prevalence rates, whereas population‐based studies often result in an underestimation of prevalence [7].

The clinical evaluation criteria for pSS have been continuously refined through ongoing academic consensus‐building efforts. As an autoimmune disease characterized by glandular symptoms, the diagnostic criteria for pSS have undergone significant evolution. It is noteworthy that related symptoms exhibit a relatively high prevalence in the general population. An epidemiological survey conducted in Sweden revealed that the incidence of xerostomia in individuals aged 50–75 years ranges between 4.6% and 22.3%, with notable variations based on gender and age [8]. Furthermore, a study focusing on women's health demonstrated that the prevalence of dry eye syndrome falls within the range of 5.7%–9.8%, showing an increasing trend with advancing age [9].

In 2016, the American College of Rheumatology (ACR) and the European League Against Rheumatism (EULAR) jointly introduced a new set of diagnostic criteria. This updated framework places greater emphasis on the significance of objective signs of dryness and increases the emphasis on immunological markers, thereby allowing patients with mainly systemic extraglandular symptoms to be included [10]. Research data from 2017 indicate that the diagnostic sensitivity of these criteria reached 95.4%, successfully identifying cases that were previously missed by the American–European Consensus Group (AECG) criteria [11, 12]. The ongoing refinement of diagnostic standards in terms of specificity and sensitivity has had a profound impact on the interpretation of epidemiological data related to pSS over the past two decades.

Studies based on three population surveys in China indicated a pSS prevalence rate of 0.45% before the application of the AECG criteria [13]. A 2005 sampling survey of one million individuals from Taiwan's health insurance database, utilizing ICD‐9 codes and rheumatologist verification, revealed an overall pSS prevalence of 0.0058%, with a female prevalence of 0.01% [14]. Although potential underdiagnosis or misdiagnosis may exist, the strength of this study lies in its derivation of data from a single, representative population. Similarly, research utilizing the French National Health Care Database, an estimated prevalence rate of 0.023%–0.032% was found among patients receiving treatments like artificial tears or immunosuppressants for SS [15].

Research on the prevalence of pSS in the United States is limited. A 2015 study conducted in a county in Minnesota reported a pSS prevalence of 0.10% based on physician diagnoses [16]. However, when stricter diagnostic criteria were applied, the prevalence decreased to 0.02%, underscoring the importance of standardized diagnostic criteria [17]. Overall, the prevalence estimates for pSS vary greatly across different research studies, primarily due to differences in diagnostic criteria and study designs. Based on the most reliable evidence from large population studies, the prevalence of pSS is thought to be in the range of 0.01%–0.05%.

The Big Data Sjögren Project is a global consortium that has gathered information from thousands of pSS patients in Europe, North America, and Asia. Statistical data show that pSS predominantly affects women (93.2%), with a stable gender distribution ratio across regions, where female patients outnumber males by 14‐fold [18]. Updated data from 2021 confirm that this female predominance remains unchanged [19]. A 2020 study demonstrated that male patients exhibit higher disease activity, with an EULAR Sjögren's Syndrome Activity Index (ESSDAI) score of 8.1 compared to 6.0 in females [20]. Male patients are more frequently affected by peripheral and central nervous system involvement, lymphadenopathy, and interstitial lung disease (ILD) [21], corroborating earlier findings from smaller studies that showed a greater incidence of extra glandular involvement in male pSS patients [22].

Recent findings indicate that the female‐specific X‐inactive specific transcript (XIST) long noncoding RNA serves as a unique abundant source of Toll‐like receptor (TLR) 7 ligands and a driver of IFN in systemic lupus erythematosus (SLE). XIST RNA stimulates plasmacytoid dendritic cells (pDCs) to produce IFN‐α in a TLR7‐dependent manner, and its absence diminishes the ability of all cell RNAs to activate TLR7. XIST levels are elevated in the white blood cells of SLE females, correlating positively with disease activity and IFN signatures compared to those in control groups. The function of XIST RNA as a female gender‐specific danger signal in the gender bias of SLE has been elucidated [23]. Risk alleles for SLE and pSS are present in both *CXorf21* and SLC15a4 [24]. Their complex affects lysosomal pH, with *CXorf21* expressed more in female than male cells, leading to enhanced TLR7 signaling and increased IFN production [24]. This sex‐based difference may underlie the gender bias in SLE and pSS [25].

### 2.2 Risk Factors for SS

**FIGURE 1:**
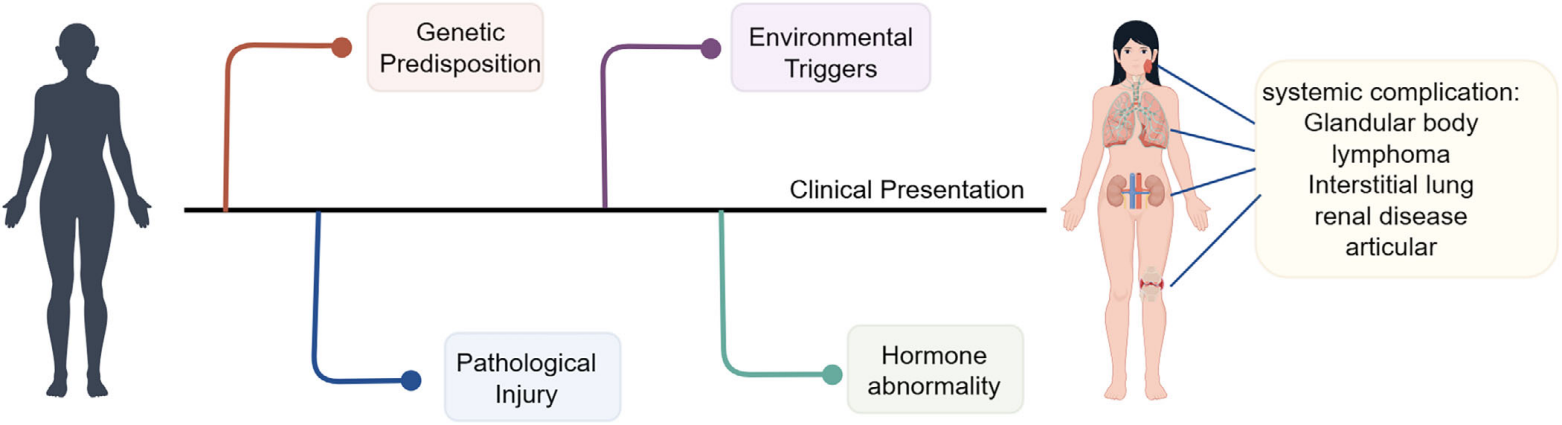
A brief view of possible etiologic events preceding the diagnosis of desiccation syndrome. Created with www.figdraw.com.

Currently, there is no consensus on the risk factors for pSS, although research has made some progress (Figure 1) [26]. Existing data from four case–control studies demonstrate that smokers have a lower risk of developing pSS [27, 28, 29], which contrasts with findings on former smoking, as no association was observed between former smoking and pSS risk [26]. However, this negative correlation may stem from reverse causality rather than a true protective effect. Statistical analysis confirms that both familial autoimmune disorders in immediate relatives [28, 30] and negative stress experiences significantly raise the probability of pSS onset [31, 32]. Adherence to a Mediterranean diet may reduce pSS risk, potentially due to its anti‐inflammatory properties [33]. Current conclusions are mainly drawn from case–control studies, a methodology that may introduce recall bias while providing relatively limited evidence.

#### 2.2.1 Hormone Abnormality

Estrogen, akin to its role in other autoimmune disorders [34], influences the development and advancement of pSS by modulating critical immune pathways, thereby potentially elevating disease risk in genetically susceptible women [35, 36]. Research indicates that estrogen exposure may exert a protective influence against pSS, whereas lower female hormone levels could be tied to the condition [3]. This hypothesis is consistent with the epidemiological patterns of pSS, as the reduction in estrogen levels during menopause is associated with increased acinar cell apoptosis [37]. In murine models, estrogen deficiency leads to pronounced autoimmune lesions in the salivary and lacrimal glands, which can be ameliorated through estrogen supplementation [38]. Estradiol, the predominant form of estrogen, has been shown to inhibit subcellular damage and confer protection to the sublingual gland [39]. In patients with pSS, the salivary gland (SG) epithelial cells (SGECs) show significantly decreased sensitivity to 17β‐estradiol [40]. Large‐scale follow‐up studies have identified an association between estrogen replacement therapy and increased incidence of dry eye syndrome [41]. Additionally, the hypothalamic–pituitary–adrenal axis, gonadal axis, and thyroid axis may also play contributory roles in the pathogenesis of pSS [37, 42, 43, 44]. Nevertheless, these factors may vary due to geographic and ethnic differences and may not apply to all patients.

#### 2.2.2 Viral Infection

Viral infections and other immune triggers might contribute to the development of pSS by causing unusual activation of epithelial cells and immune responses [45]. Viral infection can induce SGECs to secrete chemokines, which function as chemical messengers to recruit and activate lymphocytes, thereby forming lymphocytic infiltration within exocrine glands [46]. Epstein–Barr virus (EBV) can infect the epithelial cells of the labial SGs, and its ongoing presence might trigger polyclonal B‐cell activation and autoantibody production [47]. Additionally, a high viral load of human T‐cell leukemia virus type 1 (HTLV‐1) may promote the production of transforming growth factor‐beta (TGF‐β), contributing to SG fibrosis in SS patients with anticentromere antibody positivity [48]. The immunological traits of HTLV‐1‐associated SS could differ from those of idiopathic SS [49].

Hepatitis C virus (HCV) is among the viruses most strongly linked to autoimmune conditions [50]. Epidemiological data demonstrate a significant correlation between HCV infection and SS development. Following the initial identification of 44 SS cases with pathological evidence in breast cancer and HCV patients by Haddad et al. in 1992, subsequent studies have added over 400 HCV‐related SS cases, strongly confirming their close association [51]. Meta‐analyses have revealed a substantial relationship between SS and HCV infection [52]. Studies have shown that HCV can replicate in the SGECs of patients with SS or chronic sialadenitis and disrupt the Th1/Th2 cytokine balance [53, 54]. Chikungunya virus (CHIKV) is a globally prevalent arbovirus that can induce autoantibodies and rheumatic diseases, example rheumatoid arthritis (RA) and spondyloarthritis [55, 56]. Carvalho et al. were the first to document the connection between CHIKV and pSS. Sequence analysis of the CHIKV proteome and SS autoantigens revealed extensive peptide sharing between the two, providing evidence for the idea that CHIKV might be causally associated with SS through autoimmune cross‐reactivity [57]. While antiviral therapy may assist in controlling persistent viral infections that initiate SS, it may prove less effective in managing the chronic disease state that no longer relies on the initial viral trigger.

#### 2.2.3 Genetic Factors

Genetic and epigenetic influences are crucial in determining the susceptibility and phenotypic traits of pSS [58, 59]. The human leukocyte antigen (HLA) region, particularly the class II genes such as DQA1, DQB1, and DRB1, represents the strongest susceptibility locus for autoimmune rheumatic diseases, including pSS [60]. Among HLA alleles, the DR2 and DR3 alleles at the DRB1 locus are consistently associated [61]. Additionally, multiple genes outside the HLA region, especially those involved in interferon signaling pathways, are also implicated in pSS pathogenesis [61]. Molecular studies indicate distinct immune gene expression abnormalities in pSS, featuring increased IL‐1β secretion in macrophages, enhanced TCL1A expression in B cells, and widespread activation of IFN response genes. Genetic marker testing also confirms significantly upregulated expression of genes including HLA‐DRB5, CTLA4, and AQP3 [62]. Gene expression analysis of SGECs revealed upregulation of interferon signaling and immune response‐related genes, including HLA‐DRA, IL‐7, and B‐cell activating factor receptors [63]. Furthermore, B lymphocytes from the SGs of pSS patients show a significant upregulation of the activation genes CD40 and CD48 [63].

Most non‐HLA genetic variants linked to pSS susceptibility are associated with the IFN pathway or interferon‐stimulated genes (ISGs) [64]. Research has discovered single nucleotide polymorphisms (SNPs) in ISGs such as IRF5, STAT4, IL12A, and OAS1 as risk factors for pSS development [65]. Additionally, polymorphisms in genes including CD28, CTLA4, PTPN22, TNF‐α, IL‐10, and CXCR5 have also been established as risk factors for disease progression [66, 67]. Other SNPs (e.g., GTF2I, MBL2, and NOTCH4) are related to pSS and other autoimmune conditions, including RA and SLE, although some SNPs associations still require further validation [65, 68]. Furthermore, an increased number of X chromosomes is a major risk factor for SS [69]. Epidemiological investigations reveal significantly elevated SS incidence in (47, XXX) individuals, showing nearly triple the risk compared to (46, XX) females and an even more pronounced difference relative to (46, XY) males [70]. Furthermore, researchers have detected triplication of the terminal segment on the X chromosome short arm in (47, XXX) cells from a mosaic SS patient (45, X/46, XX/47, XXX) [71]. These findings provide valuable insights for future SS research.

The risk of pSS could be heightened by various genetic polymorphisms, their functional impacts remain unclear, necessitating further functional studies and research into gene interactions to elucidate their roles.

### 2.3 Risk of Lymphoma in SS

The development of lymphoma associated with SS is intricate and not completely comprehended. The process is thought to involve multiple steps, mainly driven by the overactivation of B cells as a result of ongoing inflammatory antigen stimulation [72]. Common lymphoma complications in SS patients include hyperplastic lymphoma and non‐Hodgkin lymphoma (NHL), particularly SG mucosa‐associated lymphoid tissue (MALT) lymphoma. Approximately 5% of SS patients develop lymphoma, facing a 10.5–48‐fold increased risk compared to the healthy population. Recent studies further support this conclusion: Saleh et al. reported that the prevalence of NHL among SS patients in Florida was 2.6%, indicating a 7.4 times higher risk than that of the general population [73]; Treppo et al. confirmed that the risk of NHL in SS patients is 3.84 times higher than healthy individuals [74]; and a large systematic review indicated that individuals with SS have a 13.7 times greater risk of developing NHL than the general population [75].

HarmonicSS's cloud computing infrastructure and AI models have recently pinpointed four biomarkers with enhanced predictive value for NHL in SS: salivary gland epithelium (SGE), cryoglobulinemia, the existence of rheumatoid factor (RF), and reduced C4 levels [76]. Further development of AI models is expected to improve the analysis of SS‐related lymphoproliferation risk. Imaging techniques, particularly widely available ultrasound, have become the primary tool for evaluating SG lesions in patients with parotid swelling (PSW) or high lymphoproliferation risk [77]. SG biopsy remains essential for diagnosing SS‐associated lymphoma, and a recent US study confirmed the safety, patient approval, and diagnostic precision of ultrasound‐guided core needle biopsy (US‐CNB) in 30 SS patients suspected of lymphoma [78].

Although minor SG biopsy retains value for SS diagnosis, it is insufficient for diagnosing associated lymphoma. In the future, refining existing diagnostic tools (e.g., ultrasound) and developing new AI models will enable better risk stratification for SS‐related lymphoproliferation, improve patient management, reduce unnecessary medical procedures, and ultimately prevent overdiagnosis.

### 2.4 SS Associated With Connective Tissue Diseases

SS is a phenotypically diverse autoimmune disease that can manifest as pSS or be associated with other connective tissue diseases (CTDs), such as SLE, RA, and systemic sclerosis (SSc). Research utilizing the French National Health Care Database revealed that among CTDs associated with pSS, SLE for 28%, RA accounts for 53%, and SSc for 13% [15]. Diagnosing SS is difficult because there are no specific symptoms or markers for it. The 2002 AECG criteria and the 2016 ACR/EULAR classification criteria serve as the basis for making a diagnosis [79]. An analysis of 237 SLE patients based on these criteria revealed SS prevalence rates of 23% according to AECG 2002 and 35% according to ACR‐EULAR 2016. Predictive factors for SS in individuals with SLE include glandular dysfunction, focal lymphocytic sialadenitis, and anti‐Ro antibodies [80]. The main benefit of the ACR‐EULAR 2016 criteria lies in their ability to facilitate early diagnosis and identify the overlap between SLE and SS. Individuals with SLE who also have SS exhibit the following characteristics: relatively advanced age of onset [81], high incidence rates of Raynaud's phenomenon and articular symptoms, along with high autoantibody positivity (particularly anti‐SSA/SSB antibodies), while oral ulcer symptoms are also more frequently observed [82]. Disease classification criteria are primarily created for clinical research, not diagnosis, and should be applied flexibly in clinical practice, considering each individual case.

The incidence of SS in RA varies significantly based on geographic region and the design of the study. A large US registry study revealed that 7870 RA patients were concurrently diagnosed with SS, and the prevalence of SS increased with the duration of RA. RA with SS is associated with seropositivity, more severe RA, extra‐articular manifestations, and comorbidities [83]. In the prospectively collected Paris‐Saclay cohort, patients were identified, and the study found that joint pain, arthritis, and pulmonary involvement were more common in the SS/RA group. Additionally, compared to the pSS group, the SS/RA group exhibited elevated ESR levels and RF positivity, although there was no significant difference in lymphoma incidence [84]. Ultimately, patients with RA and SS exhibit more complex clinical features and higher disease activity, necessitating closer monitoring and individualized treatment strategies.

Symptoms of dryness, like dry mouth and dry eyes, are very common in SSc patients, with occurrence rates between 63% and 71% [85]. A study involving 118 patients demonstrated that 63% of individuals exhibiting sicca symptoms and undergoing SG biopsy were diagnosed with SSc and SS [86]. Research involving 1132 SSc patients from Italian and French cohorts identified that 12% had concomitant SS, establishing it as the most prevalent autoimmune disease linked with SSc. SSc patients with SS showed a reduced occurrence of diffuse SSc, pulmonary fibrosis, and pulmonary hypertension compared to those without SS, but higher incidences of limited cutaneous SSc, arthritis, hypergammaglobulinemia, anticentromere antibodies, antibodies to SSA/SSB and RF [87, 88]. The cross‐sectional study of 1261 patients with SS revealed that individuals with anticentromere antibodies experienced more severe exocrine gland dysfunction and heightened SG inflammation, but had reduced fibrosis [89]. Although SSc patients were excluded from this study, the increased prevalence of Raynaud's phenomenon, sclerodactyly, and telangiectasia in these patients is consistent with the overlapping features of SS and SSc features.

## 3 Classification Criteria

The progression of pSS disease is fundamentally driven by the appearance of abnormal autoreactive B cells, which results in the generation of autoantibodies and the creation of immune complexes [90]. Expert working groups have established classification criteria for SS, with the latest criteria and disease activity scores primarily aimed at research, yet they are also practical in routine clinical practice. The 2016 ACR/EULAR consensus classification criteria are determined by factors like anti‐SSA/Ro antibody presence, focal lymphocytic sialadenitis focus score, ocular staining score, Schirmer's test, and unstimulated salivary flow rate, classify individuals with sicca symptoms using a scoring system [91, 92]. With a sensitivity of 96% and a specificity of 95%, these criteria are well‐suited for diagnostic validation and clinical trials [91, 93]. Although primarily designed for research, they also provide practical utility in clinical practice.

## 4 The Molecular Pathogenesis of SS

### 4.1 Salivary Gland Epithelial Cells in SS

**FIGURE 2:**
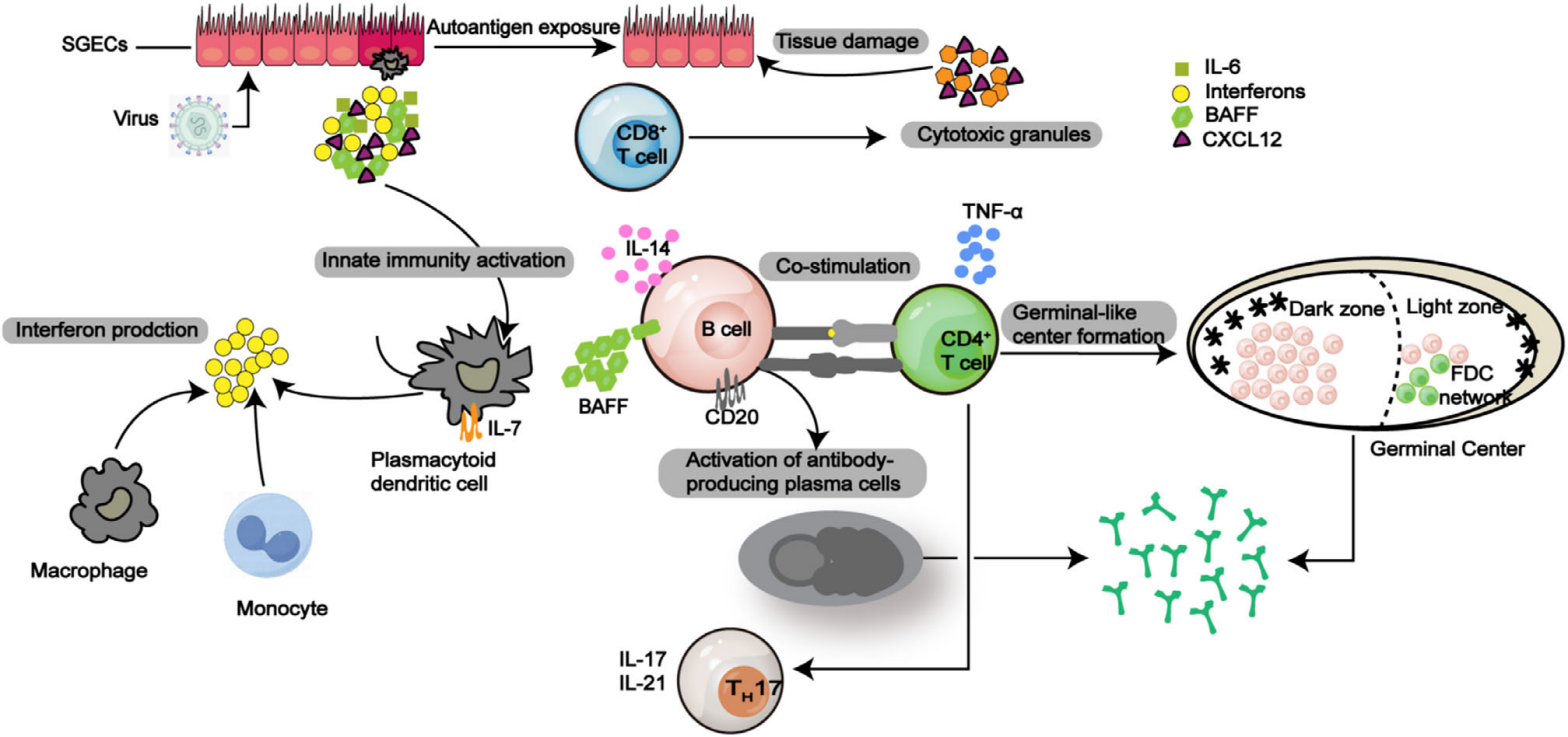
Sjögren syndrome pathogenetic model. In genetically susceptible individuals, viral infection is thought to trigger activation of SGECs, which leads to activation of the innate immune system and ultimately to interactions between innate and adaptive immune cells. Activated SGECs can act as antigen‐presenting cells and produce a specific cytokine milieu, including IL‐6 (dark green squares), IFN‐I (yellow circles), CXCL12 (purple triangles), and BAFF (light green hexagons). In addition, overexpression of IFN‐I by plasmacytoid dendritic cells promotes B‐cell differentiation and survival, sges injury triggered by exogenous triggers and sustained by activated CD8^+^ T cells and immune complexes, leading to chronic release of self‐antigens, which in turn stimulates innate immune activation in a vicious cycle. B cells, monocytes and macrophages, innate‐like lymphocytes and T helper 17 (TH17) cells, as well as a variety of cytokines and chemokines are involved in the development of the disease.

For those with genetic susceptibility, environmental elements may target SGECs, leading to the activation of the resting epithelium and subsequent aberrant immune responses (Table 1). Studies demonstrate that microbial activation of TLR signaling pathways (involving receptors such as TLR3, 7, 9) in SGECs induces the release of inflammatory mediators, initiating local inflammatory responses. During this process, SGECs upregulate CD40 and cellular adhesion molecules, establishing the molecular basis for immune cell infiltration (including dendritic cells and T/B lymphocytes) at lesion sites (Figure 2) [106].

**TABLE 1:** The molecular pathogenesis of SS.

Cell type	Function	References
Salivary gland epithelial cells	Release of proinflammatory cytokines, key to immune response initiation and propagation.	[94, 95, 96]
Neutrophils	Overactivation of IFN‐I leads to abnormal neutrophil activation, the formation of NETosis.	[97, 98]
Plasmacytoid dendritic cells	Aberrant activation of pDCs and excessive production of IFN‐I.	[99, 100]
B cells	Hyperactivation causes the glands to swell and the creation of anti‐SSA and anti‐SSB autoantibodies.	[101]
T cells	Th1, Th17, and Tfh in the infiltration of glands, leading to epithelial cell damage and perpetuating inflammation by secreting inflammatory mediators.	[102, 103, 104, 105]

In the SG, the main type of cell is the epithelial cell, known for producing saliva [107]. The initiation and continuation of the immune response in SS heavily rely on SGECs. The onset of SS is considered to begin with disrupted exocrine gland homeostasis [94]. SGECs secrete a variety of lymphoid chemokines, such as CXCL12 and CXCL13, that play a role in attracting inflammatory lymphocyte populations early in the inflammatory process [95, 108]. Additionally, cytokines that promote inflammation, such as IL‐1, IL‐6, and TNF‐α, along with survival factors like BAFF and IL‐7, are released by SGECs, which contribute to the inflammatory environment and regulate the inflammatory response within the tissue [95, 96].

Specifically, CXCL12 and IL‐6 expression has been demonstrated to draw and maintain a group of CXCL12^+^ BCL‐2^+^ plasma cells that generate autoantibodies typically linked with pSS, like anti‐Ro/SSA and anti‐La/SSB [109]. The proinflammatory conditions present in SS impairs SGECs function and causes their demise [63, 110]. According to the study revealed that SGECs exhibit antigen‐presenting capabilities, boost T cell growth and activation in SS, resulting in glandular damage [111], and B cells are activated and differentiated in SS [101]. A group of protein molecules known as TLRs is connected to innate immunity, and the upregulation of TLR expression by SGECs in SS, along with TLR binding to receptors, triggers the creation of type I IFN (IFN‐I) and additional cytokines. These results indicate that SGECs could be involved in the pathogenesis of SS [112].

Lysosome‐associated membrane protein 3 (LAMP3) is overexpressed in individuals suffering from SS. Aberrant expression of LAMP3 in SGECs can be induced by enhanced IFN‐I response, while LAMP3 promotes IFN‐I production through upregulation of ectopic TLR‐7, creating a positive feedback that maintains its misexpression and exacerbates the generation of IFN‐I in the SGs, which is crucial in the development of SS pathophysiology [113]. Acinar cells exposed to IFN‐γ in vitro demonstrated alterations in tight junction function and increased epithelial permeability [114]. SG tissue from patients with pSS showed similar changes in tight junction components [115]. Furthermore, IFN‐γ was found to induce apoptosis in SGECs cultures, indicating that IFN‐γ accumulation from Th1 cells in exocrine glands could cause harm to epithelial cell and lower salivary secretion [116].

Here, epithelial cells play an active role in starting and sustaining autoimmune responses through various mechanisms, though the reason for their ongoing dysregulation is still unclear.

### 4.2 The Presence of Immune Cells in SS

#### 4.2.1 Neutrophils in pSS

Neutrophils are not only primary responders to pathogen invasion but are also closely associated with the development of autoimmune conditions such as SLE [117], RA [118], and ANCA‐associated vasculitis (AAV) [119]. Research indicates that a key characteristic of pSS patients is an IFN signature, marked by notably increased levels of IFN‐I in both plasma and labial glands (Table 1) [120]. Overactivation of IFN‐I leads to abnormal neutrophil activation [97], and neutrophils are essential for fighting infections and preserving tissue health.

In SS patients, excessive stimulation of IFN‐I induces a hyperactive state in neutrophils, thereby initiating an inflammatory cascade. Through the process of NETosis, neutrophils release neutrophil extracellular traps (NETs) consist of chromatin, histones, proteases, cytoplasmic proteins such as MPO and NE [98]. Research by Yunyun Fei's team revealed that in SS patients plasma levels of MPO and cf‐DNA were notably higher than in healthy individuals, and neutrophils from pSS patients showed increased secretion of cf‐DNA and MPO, suggesting that NETs could be involved in SS pathogenesis [97]. Furthermore, the activation of IFN‐I is associated with increased ROS production in neutrophils of pSS patients, and excessive ROS may lead to tissue damage in salivary and lacrimal glands, thereby promoting disease progression [121].

Accordingly, excessive activation of IFN‐I in neutrophils may result in aberrant immune system activation via upregulation of NETosis and ROS, potentially contributing to SS. More in‐depth studies are required to clarify the exact mechanism.

#### 4.2.2 Plasmacytoid Dendritic Cells in pSS

**FIGURE 3:**
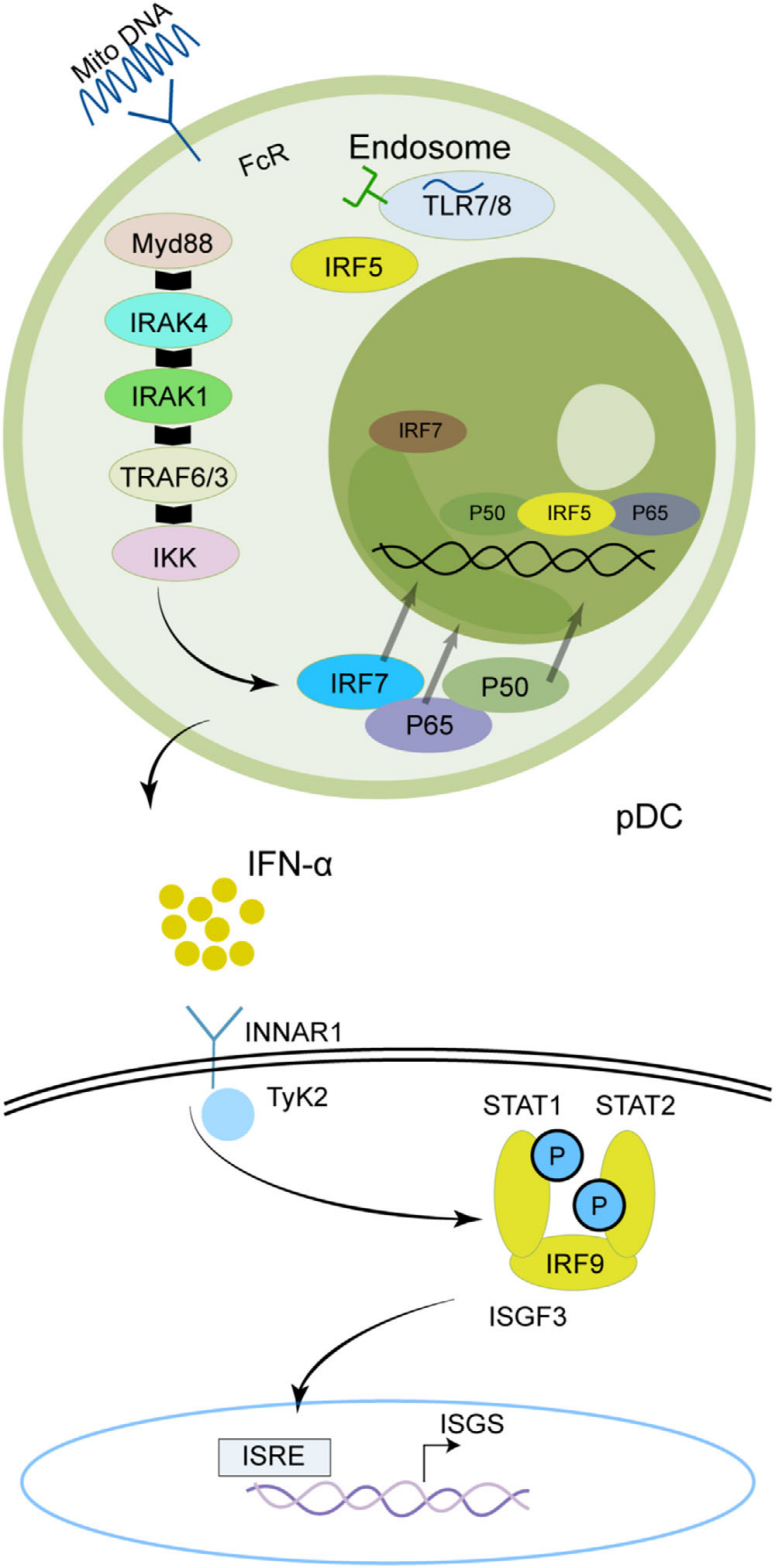
Nucleic acid sensors and downstream signaling pathways induce type I IFN production. Cytoplasmic sensors for RNA (RIG‐1) and DNA (cGAS) signal through the adapters MAVS and STING, respectively, and activate the kinases TBK1 and IKKε, as well as downstream IRF3 (left). IRF3 translocates to the nucleus, cooperates with NF‐κB, and drives IFN‐β transcription in a variety of cell types including macrophages and epithelial cells. In pDCs producing large amounts of IFN‐α, nucleic acid‐containing immune complexes are endocytosed and delivered to the nuclear endosomes via the Fc receptor (FcR), which then activates TLR sensors in RNA (TLR7, TLR8) or DNA (TLR9) (right). These endosomal TLRs signal through the junction MyD88 and activate the IKK kinase complex, which in turn activates downstream transcription factors, including IRF7 and the NF‐κB subunits p50 and p65. IRF5 is activated by an unknown mechanism. These transcription factors translocate to the nucleus and drive IFN‐α transcription.

The primary functions of pDCs in the immune system involve pathogen recognition and the generation of IFN‐I (Table 1 and Figure 3). Notably, pDCs are the most effective IFN‐I producers, with their capacity for IFN‐I production upon activation exceeding that of other cell types by a factor of 1000 [99, 100].In SS pathogenesis, abnormal pDC activation and excessive production of IFN‐I are linked to immune system dysregulation and the disease progression. Experimental studies have shown that early depletion of pDCs can mitigate the severity of autoimmune disorders [122, 123, 124, 125].

pDCs represent a distinct category of sentinel cells that are adept at detecting pathogen‐derived nucleic acids and responding with the rapid and robust production of IFN‐I [126]. In pDCs that produce substantial quantities of IFN‐α, nucleic acid‐containing immune complexes are internalized via Fc receptors (FcRs) and subsequently transported to endosomes, where they activate TLRs such as TLR7, TLR8, or TLR9. Through the adaptor protein MyD88, these TLRs start a signaling cascade that activates the IKK complex, ultimately leading to the stimulation of transcription factors, including IRF7 and NF‐κB, thereby enhancing the transcription of IFN‐α. The inhibitory mechanisms exerted by pDC surface receptors, such as BDCA2, CD123, and ILT7, on IFN‐α production remain inadequately elucidated [127]. Artesunate (ART), a first‐line antimalarial drug [128], has recently been shown to alleviate SS through suppression of the TLR‐MyD88‐IRF7 signaling pathway and downregulating IFN‐α in pDCs, this finding has been dually validated in clinical specimens and NOD mouse experiments [128].

Furthermore, it has been suggested that neutrophil death (the NETosis process), as a key factor in the chronic activation of pDCs by releasing extracellular DNA and proteins that can be recognized by pDCs, thereby stimulating the production of IFN‐I [117, 129, 130, 131]. Excessive production of IFN‐I may trigger B lymphocytes to produce autoantibodies, resulting in immune system attacks on tissues, particularly those related to saliva and tear glands, further exacerbating the pathological process of SS.

#### 4.2.3 B Cells in pSS

Research indicates that the characteristic microenvironment in lesioned tissues of pSS significantly promotes prolonged B‐cell activation; however, recent investigations have highlighted intrinsic factors that may contribute to this overactivation (Table 1). In SS patients, aberrant B‐cell activation within exocrine glands leads to glandular tissue swelling and triggers the generation of anti‐SSA and anti‐SSB autoantibodies [101].

In SS, IFN‐I enhances antibody production by stimulating B‐cell activation, proliferation, and differentiation, thereby driving autoimmune responses targeting the salivary and lacrimal glands [132]. IFN can amplify TLR signaling, which is crucial for T‐cell‐independent B‐cell responses. TLR can detect both exogenous pathogens and endogenous self‐antigens, particularly nuclear antigens released from apoptotic cells. These nuclear antigens are believed to be pivotal in disrupting immune tolerance and initiating autoimmunity through the coordinated activation of the BCR and TLR signaling pathways [133, 134]. Bekeredjian‐Ding et al. demonstrated that IFN‐I produced by pDC enhances B cells responsiveness to TLR7 by selectively increasing TLR expression. In the context of IFN‐I stimulation, activation of TLR7 induces the proliferation of diverse B cells populations and drives their differentiation into immunoglobulin‐producing plasma cells [135]. Brauner et al. have consistently observed that naive B cells derived from patients with pSS exhibit a hyperresponsive phenotype typified by upregulation of numerous immune‐related genes, particularly those involved in endosomal TLR signaling pathways. Furthermore, upon stimulation with endosomal TLR ligands, these hyperreactive B cells demonstrate an augmented capacity for class switching and differentiation into plasma cells [136].

In summary, IFN‐I plays a pivotal regulatory role in the aberrant activation of B cells in SS through the TLR signaling pathway. Future research could further explore therapeutic strategies targeting the TLR signaling pathway to improve the clinical management of SS.

#### 4.2.4 T Cells in pSS

In individuals with SS, various immune cells infiltrate the exocrine glands. T‐cell subsets are pivotal in the autoimmunity associated with SS, as they control intricate immune reactions. Specifically, during the initial phases of the disease, Th1 and Th17 cells invade the glands, leading to epithelial cell damage and perpetuating inflammation through the release of inflammatory mediators [102, 103]. During the later stages, the glands are infiltrated by T follicular helper (TFH) cells and B cells, with TFH cells supporting the differentiation of B cells and the generation of antibodies (Table 1) [104, 105]. Regulatory T cells (Tregs) might be involved in preserving immune balance and controlling the processes that prevent the loss of self‐tolerance [137]. The significant presence of T cells and various cytokines in the exocrine glands of SS animal models highlights the essential role of T cells and their cytokines in SS pathogenesis [138].

In individuals of pSS, CD4^+^ T lymphocytes and their associated cytokines, particularly IFN‐γ, are prevalent in both the affected organs and peripheral blood [139]. Research indicates that most CD4^+^ T cells in the inflamed glandular tissues of pSS patients produce IFN‐γ, exhibiting a Th1 cell phenotype, and IFN‐γ‐producing Th1 cells are predominantly found within lymphocyte infiltrates [140]. In SS patients, the lacrimal glands contain a greater number of CD4^+^ T cells than CD8^+^ T cells, which have a tissue‐resident memory phenotype. In SS mouse models, the submandibular glands are dominated by CD8^+^ T cells, which show a notable rise in IFN‐γ production. Knockout of CD8a or IFN‐γ inhibits SS progression, reduces the infiltration of CD8^+^ T cells, and lessens glandular damage. Alleviating pathological effects by depleting CD8^+^ T cells after the disease starts offers a potential direction for future SS therapies [141]. Research indicates that CD8^+^ T cells have a distinct phenotype and potential pathogenic role in SS, contributing to acinar damage in exocrine glands [141, 142]. Additionally, SS patients show an expansion of IL‐17‐producing CD4^−^CD8^−^ T cells in their peripheral blood, which infiltrate the SGs, whereas CD4^+^CD8^+^ double‐positive T cells could provide protection [143]. In the aly/aly spontaneous SS mouse model, Kurosawa et al. identified elevated levels of CXCR4 and CXCR12 expression, leading to aberrant T‐cell infiltration [144]. The expression of specific nuclear matrix‐binding domain‐binding protein 1 (SATB1), is unique to T cells within mature hematopoietic cells. SATB1‐specific knockout (SATB1cKO) mice exhibit a spontaneous decrease in salivary secretion and a decrease in salivary vesicles, indicating that T cells play a crucial role in the development of SS [145].

### 4.3 The Role of Signaling Pathways in the Development of SS

#### 4.3.1 Signaling Pathway of the IFN in SS

The IFN‐I system exhibits dysregulation across multiple autoimmune diseases, including SLE, pSS, RA, SSc, and dermatomyositis (DM), as demonstrated by genetic, transcriptomic, and immunological evidence (Table 2) [146, 153]. These diseases share common pathogenic mechanisms that contribute to autoimmunity, wherein IFN‐I activity plays a pivotal role, and they exhibit considerable overlap in their clinical manifestations [154]. The significance of the IFN‐1 pathway is notably pronounced in desiccation syndrome, exhibiting distinct correlations with SSA antibodies and HLA class II DR 3 and DR 2 haplotypes, alongside the IFN gene signature [155]. These haplotypes are also linked to elevated IFN signaling in conditions such as SLE, RA, and SSc [156–158]. The IFN signaling pathway is a core component of SS, and its disruption often results in tissue damage and inflammation, with the SGs being primarily affected [146, 147].

**TABLE 2:** Signaling pathways in the development of SS.

Pathogenesis	Function	References
Type I IFN	The IFN signaling pathway is a core component of SS, and its dysregulation often causes tissue damage and inflammation, primarily impacting the salivary glands.	[146, 147]
Toll‐like receptor	Stimulation of SGECs promotes antigen presentation and positively correlates with salivary gland inflammatory marker levels, and also stimulates immature B cells and promotes plasma cell differentiation and class switching.	[148, 149, 150]
NF‐κB	NF‐κB is central to the downstream signaling of TLR pathways, also modulates inflammasome activity, being crucial in the development of inflammatory diseases.	[151]
PI3K/Akt/mTOR	enhances TLR4‐mediated NF‐κB transactivation via PI3K/Akt signaling, thereby inducing inflammation, while concurrently inhibiting TLR‐mediated activation of downstream transcription factors and the formation of inflammatory mediators.	[152]

**FIGURE 4:**
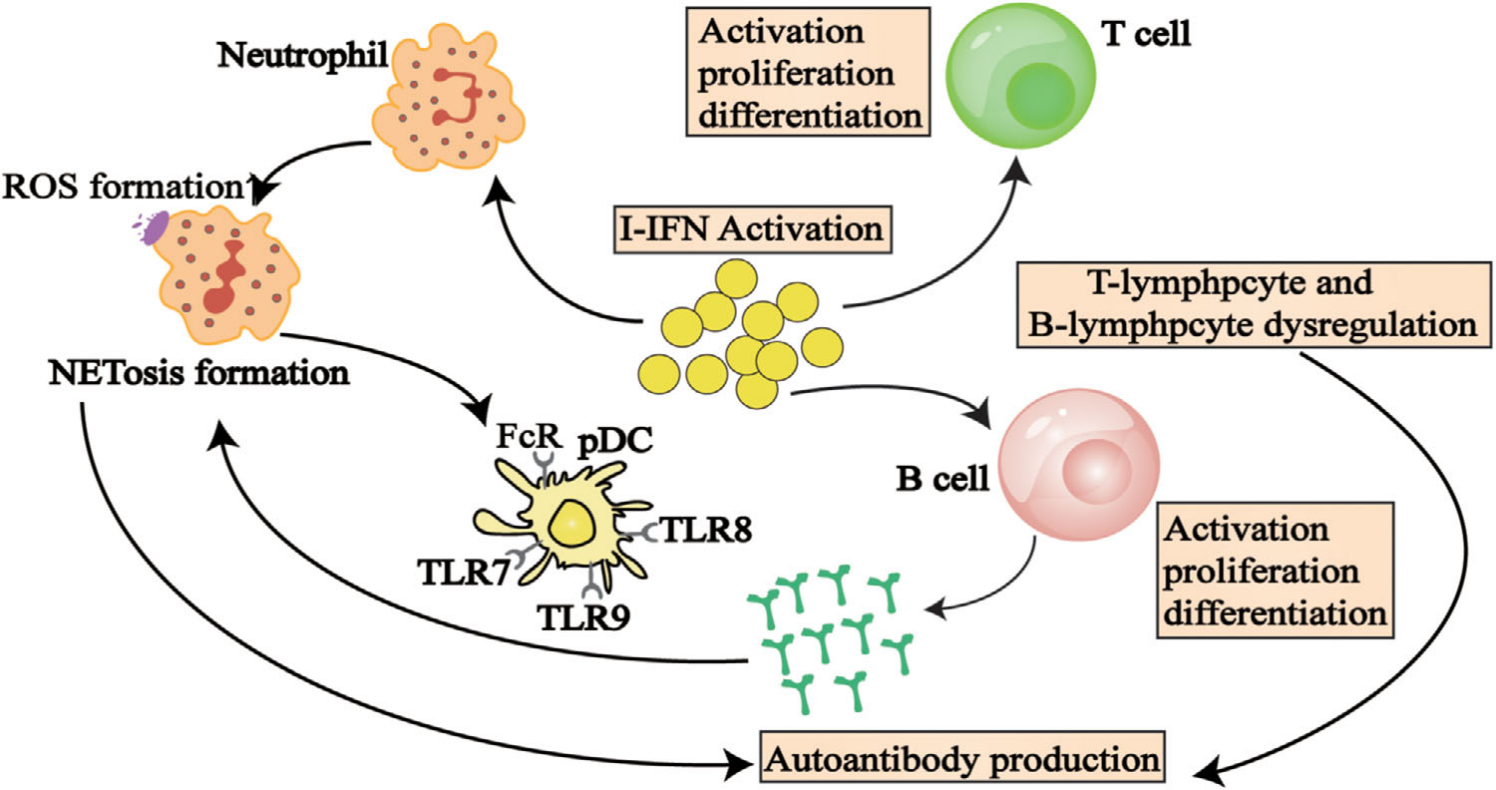
The involvement of IFN‐I‐activated immune cells and salivary gland epithelial cells (SGECs) in the pathogenesis of SS.

Interferons are divided into three categories according to their receptors: type I (IFN‐α, IFN‐β, IFN‐ω), type II (IFN‐γ), and type III (IFN‐λs) [159]. IFN‐I production can occur in different types of cells (Figure 4), but the primary pathway for blood cells to secrete IFN‐I in response to viruses is through pDCs inducing nucleic acids via TLR7 and TLR9. IFN‐I binds to the cell surface receptor IFNAR activates JAK1 and TYK2, leading to the phosphorylate of STAT1 and STAT2, forming the STAT1‐STAT2‐IRF9 (ISGF3) complex [159]. This complex binds to ISRE, upregulating ISGs and inducing downstream target gene expression. Additionally, IFNs can activate signal transduction through STAT1 homodimers, and STAT3, STAT4, and STAT5 may also be activated. Transcription factors of the IRF family interact with STATs to regulate IFN characteristics. IFNs can also elicit diverse cellular effects through alternative signaling pathways independent of JAK/STAT activity, such as the MAPK and PI3K pathways [160].

In SS patients, the interaction between B‐cell activation and the IFN pathway is evident, with B cells capable of inducing IFN production, thereby promoting autoantibody generation [161, 162]. The involvement of the IFN‐I system in the development of SS is well‐established, as evidence by increased levels of IFN‐induced genes in the SGs of pSS patients. Around 55% of pSS patients exhibit IFN signaling in peripheral blood, which correlates with increased disease activity, specific autoantibodies (particularly anti‐Ro/SSA and anti‐La/SSB), and elevated immunoglobulin levels [132]. Studies have shown the dysregulation of miRNAs and lncRNAs in peripheral blood mononuclear cells, SG tissues, tears, and saliva of SS patients [163]. In this group of molecules, hsa‐miR‐145‐5p, shows decreased expression in SS, exhibits anti‐inflammatory properties and serves as a molecular link between IFN‐I and glandular dysfunction/inflammation by upregulating MUC1 and TLR4 Dysregulation of hsa‐miR‐145‐5p due to overactivation of the IFN‐I pathway results in heightened expression of MUC1 and TLR4, thereby driving the persistent progression of abnormal immune responses [164].

#### 4.3.2 Signaling Pathway of Toll‐Like Receptor in SS

In innate immunity, TLRs are essential mediators of inflammatory pathways, and their activation can lead to the activation of factors such as c‐Jun kinase, NF‐κB, and p38 mitogen‐activated protein kinase (p38 MAPK) [165]. In SS, TLR9 activation induces the phosphorylation of p38/MAPK and JNK over time [166]. TLR7 stimulates SGECs to promote the presentation of Ro 52‐SS‐A antigens via MHC class I [148] and shows a positive relationship with SG inflammation markers such as CXCL13, CXCR5, TNF, and LT‐α [149]. TLR7 also stimulates immature B cells, promoting plasma cell differentiation and class switching (Table 2) [150]. LAMP3 amplifies IFN‐I production and induces ectopic TLR7 expression [167]. TLR2 drives autoimmune inflammation by inducing Th17 cell pathogenicity and IL‐15 production [168]. TLR4 recognizes the ectopic occurrence of mucins MUC5B and MUC7 in the SGs of patients with SS, leading to significant increases in CXCL8, TNF‐α, IFN‐α, IFN‐β, IL‐6, and IL‐1b, thereby promoting the chronic state of SS [169]. Additionally, dsDNA viruses and Poly(I:C) stimulate SGECs via TLRs to produce BAFF, whose overexpression enhances B‐cell infiltration and induces their differentiation into GC B cells, thereby linking innate and adaptive immunity [170].

Recent research has demonstrated that calcium ion (Ca^2+^) signaling through store‐operated Ca^2+^ entry (SOCE), facilitated by stromal interaction molecules STIM1 and STIM2, in conjunction with ORAI1 Ca^2+^ channels, is crucial for salivary secretion [171]. In murine models deficient in STIM1 and STIM2 within SGs, there is a notable reduction in Ca^2+^ levels and saliva production, without any indication of lymphocyte infiltration or elevated levels of SS‐specific autoantibodies, which may be attributed to the downregulation of TLR8 expression. Increased expression of STIM1 and TLR7/8 has been observed in SG biopsies from SS patients [172]. Through high‐throughput sequencing of B cells, the importance of the TLR pathway was elucidated, and the involvement of the TLR7 signaling pathway in thrombocytopenia related to pSS was investigated. In patients with pSS‐associated thrombocytopenia, B cells showed increased expression of IL‐8 and molecules from the TLR7 pathway. Furthermore, platelet counts were found to be inversely related to the serum concentrations of IL‐1β and IL‐8 [173].

#### 4.3.3 Signaling Pathway of NF‐κB in SS

In the downstream signaling of TLR pathways, NF‐κB is pivotal. It is activated by biological stress and then relocates to the nucleus to initiates immune responses and regulates the expression of inflammatory cytokines [174]. NF‐κB also modulates inflammasome activity, playing a critical role in the progression of inflammatory conditions (Table 2) [151].

Research indicates that protein arginine methyltransferase 5 (PRMT5) alleviates B‐cell hyperactivation by negatively influencing the RSAD2/NF‐κB signaling pathway, and inhibiting PRMT5 notably relieves symptoms in pSS mice [175]. Elevated expression of epithelial–stromal interaction 1 (EPST1) in B cells of pSS patients activates the TLR9/p65/NF‐κB signaling pathway by promoting IκBα degradation, causing irregular activation of B cells [176]. The risk allele of GTF2I SNP risk allele increases GTF2I expression in SGs, activating the NF‐κB pathway, suggesting that GTF2I could be a therapeutic target for SS [177]. The presence of BST‐2 (Tetherin/CD317), in B cells from peripheral blood or SGs, promotes B‐cell proliferation and survival by modulating NF‐κB signaling [178]. Overexpression of radical S‐adenosyl methionine domain‐containing 2 (RSAD2) in CD19^+^ B cells of pSS patients can be silenced to attenuate B‐cell activity by targeting the NF‐κB pathway, offering a potential therapeutic target for pSS [179]. High mobility group box 1 (HMGB1) promotes inflammation in various diseases. In an SS model, inhibition of HMGB1 improved xerostomia triggered by SS by suppressing the HMGB1/TLR4/NF‐κB signaling pathway and upregulating AQP5 expression [180].

Studies have shown that miR‐23b‐3p can serve as a preventive measure for autoimmune diseases by focusing on SOX6 and blocking the NF‐κB signaling pathway, thereby alleviating SS symptoms [181]. In SS patients and INF‐γ‐induced SGECs, miR‐223‐3p expression is increased while ITPR3 is decreased. Knockdown of miR‐223‐3p enhances cell viability and suppresses apoptosis and inflammatory reactions via the NF‐κB signaling pathway [182]. Molecular aberrations in the NF‐κB pathway are commonly observed in thymic MALT lymphoma, suggesting that its dysregulation is a critical component of the disease's pathogenesis [183]. NF‐κB is crucial in regulating physiological inflammatory responses, further exploration of its potential clinical applications is warranted, including therapeutic interventions and the utilization of NF‐κB as an indicator for different disease types and treatment outcomes.

#### 4.3.4 Signaling Pathway of PI3K/Akt/mTOR in SS

Through analysis of publicly available transcriptomic data and bioinformatics, differential gene expression profiles in individuals with scleroderma, lupus erythematosus, and SS revealed the PI3K/Akt pathway as the most frequently enriched pathway [184]. Regulating a variety of cellular functions, the PI3K/Akt/mTOR signaling pathway is vital for processes like survival, proliferation, growth, metabolism, angiogenesis, and metastasis [185, 186]. Activation of PI3K enhances TLR4‐driven NF‐κB transactivation through PI3K/Akt pathway, thereby inducing inflammation, while concurrently inhibiting TLR‐mediated activation of downstream transcription factors and the creation of inflammatory mediators (Table 2) [152]. Vasoactive intestinal peptide (VIP) exhibits anti‐inflammatory and immunomodulatory effects, and treatment of NOD mice with VIP increases PTEN and VIP/VPAC1 receptor expression while reducing PI3K/Akt pathway activity. Knockdown of PTEN decreases the Treg/Th17 ratio and enhances PI3K/Akt phosphorylation, an effect reversed by VIP treatment [187]. In the minor SGs of SS patients, the Akt pathway is particularly activated, and mTOR may regulate SG atrophy [188]. Autophagy, which is influenced by mTOR and the PI3K/Akt signaling pathway, is a vital mechanism for maintaining acinar cell integrity during SG atrophy [189]. Further research is necessary to determine how the activation of the PI3K/Akt pathway leads to metabolic changes that affect immune responses in SS.

## 5 The Diagnosis of SS

SS is a long‐term autoimmune disorder mainly marked by dryness in the eyes and mouth due to issues with the lacrimal and SGs. It can also involve multiple organ systems, presenting with diverse clinical manifestations and varying severity among patients [90]. The main technique for diagnosing glandular issues and evaluating autoimmune activity is a biopsy of the labial SG, playing a crucial role in disease classification and stratification in clinical trials [190]. Diagnosis of SS depends on recognizing its specific clinical manifestations, SG histopathology, and autoantibody detection [191]. Two assessment tools developed by EULAR, the ESSPRI, a questionnaire for evaluating subjective symptoms, and the ESSDAI, a clinical tool for assessing systemic disease activity, has shown high validity, reproducibility, and effectiveness in monitoring changes in disease activity [192].

### 5.1 Traditional Diagnostic Criteria for SS

According to the 2016 ACR/EULAR classification criteria are relevant for patients who show at least one sign of eye or mouth dryness, as determined by AECG questions, or those with positive findings in at least one domain of the ESSDAI questionnaire, indicating a suspicion of SS [91]. A patient is diagnosed with pSS if their total score is 4 or higher based on these criteria. The score is determined by adding up five objective elements: a score of 3 points is assigned for having anti‐SSA/Ro antibodies and/or focal lymphocytic sialadenitis with a focus score of 1 or more foci per 4 mm^2^; a score of 5 or above on the OSS (or 4 or more on the van Bijsterveld scale), a Schirmer's test result of 5 mm or less in 5 min, and an unstimulated salivary flow rate of 0.1 mL per min or less, each assigned 1 point.

Serological markers characteristic of SS include antinuclear antibodies (ANAs), RF, anti‐Ro/SSA, and anti‐La/SSB antibodies [193]. Up to 85% of patients have ANA, anti‐Ro/SSA and anti‐La/SSB antibodies, which appear in 33%–74% and 23%–52% of SS patients, respectively, are regarded as crucial immunological indicators of the disease [194]. However, the isolated detection of anti‐SSA/Ro or anti‐SSB/La antibodies is not enough to diagnose SS, since these antibodies may also appear in other connective tissue disorders or in healthy people [195]. Additionally, in pSS patients, BAFFR expression is notably decreased and linked to changes in B‐cell subsets, indicating that lower BAFFR expression might be an early marker of B‐cell involvement and could have diagnostic significance [196].

### 5.2 New Insights Into SS Diagnosis

The addition of new biomarkers or the use of salivary gland ultrasonography (SGUS) could improve the effectiveness of existing diagnostic criteria for SS [197]. SGUS has been widely utilized in SS diagnosis, and the OMERACT scoring system facilitates the objective evaluation of ultrasound findings, improving the specificity of classification criteria. Studies suggest that factors such as psychological stress, cardiovascular disease risk, and lymphoma risk contribute to more precise identification of high‐risk patients [198]. When the SGUS OMERACT score (cut‐off ≥ 2) is integrated into the ACR/EULAR criteria, diagnostic accuracy remains robust (AUC 0.974), with a sensitivity of 96.4% and specificity of 86.5% [199]. The combination of SGUS OMERACT scoring with the ACR/EULAR criteria demonstrates excellent performance in predicting clinical SS diagnosis, thereby expanding diagnostic options for suspected patients.

## 6 Treatment of SS

Despite advancements in the understanding of the pathophysiology of SS, its treatment continues to be predominantly empirical and focused on alleviating symptoms, primarily through the use of saliva and tear substitutes, analgesics, and glucocorticoids (GCs) for managing pain and fatigue (Table 3). The effectiveness of immunosuppressive drugs, primarily used to reduce GC usage, lacks substantial evidence. Initial trials utilizing repurposed therapies from other autoimmune rheumatic diseases have often not demonstrated substantial effectiveness in SS, rendering its management in clinical practice particularly challenging.

**TABLE 3:** Progress in clinically relevant drug trials.

Designation	Mechanisms	Developments
Anifrolumab	An antibody of the monoclonal type aimed at the IFNAR	In Phase II trials (NCT05383677)
Filgotinib	Reduced IFN‐I signature activity in patients with SS	In Phase II trials (NCT03100942)
Tofacitinib	A selective JAK1 and/or JAK3 inhibitor alleviates ILD due to its anti‐inflammatory and antifibrotic effects	In clinical trials (NCT 04496960)
Leflunomide‐hydroxychloroquine	ESSDAI scores correlated with alterations in three IFN‐induced proteins	A new multicenter clinical trial
Belimumab/rituximab	Enhance peripheral memory B cells, diminish naïve, activated, and plasma B‐cell subsets, enhance the selectivity of B‐cell reconstitution	In Phase II trials (NCT02631538)
Epratuzumab	Improvement in disease activity accompanied by lower B‐cell counts and IgM levels	A 2018 clinical trial
Iscalimab	An innovative anti‐CD40 monoclonal antibody, reduction of the ESSDAI scores	Clinical trial (NCT02291029)
Tibulizumab	An antibody with dual antagonistic properties against BAFF and IL‐17A	Phase I trials (NCT02614716)
JH013 injection	An antibody of human origin that aimed at the BAFF receptor	Phase I clinical trial
Telitacicept	A novel, fully human TACI‐Fc fusion protein that attaches to both BAFF and APRIL	Phase III clinical trial
Iguratimod	The NF‐κB activity inhibitor	Phase IIb trials (NCT02962895)
Ianalumab (VAY736)	Directly killing B cells or by blocking the BAFF receptor and further interfering with the signaling pathway	Phase III study
Deucravacitinib	TYK2 inhibitor, interferes with IFN‐I signaling and with IL‐23/IL‐12 signaling	Phase III study
Branebrutinib/remibrutinib	BTK‐targeted therapies	Phase III trial (NCT04186871)/Phase II studies
Nipocalimab/efgartigimod alfa	Targeting the neonatal FcRn to reduce circulating IgG antibodies	Phase II trial (NCT04968912)/(NCT05817669)
Low‐dose interleukin 2	Restored immune homeostasis by enhancing regulatory T cells and CD24^hi^ CD27^+^ B cells	NCT02464319
Ustekinumab	A human monoclonal antibody targeting the shared p40 subunit of IL‐12 and IL‐23	NCT04093531
Venanprubart	A BTLA‐targeted agent, BTLA suppresses both TCR‐mediated proliferation and cytokine production in T cells, while concurrently inhibiting B‐cell proliferation	Phase II clinical trial is currently assessing
IBI355	A CD40L antibody, work by blocking the binding of CD40L to CD40, which results in the suppression of abnormal immune cell activation and a decrease in inflammatory responses.	Phase I clinical trial
Isocalimab (CFZ533)	A fully human IgG1 anti‐CD40 monoclonal antibody	Phase II trials (NCT04541589)
Dazodalibep	A novel nonantibody TN3 fusion protein functioning as a CD40L antagonist	Phase III trials (NCT06104124/NCT06245408)
Abatacept	Modulate T‐cell costimulation in SS by selectively preventing CD28 interactions with CD80/CD86	The ASAP‐III trial
Prezalumab	A humanized anti‐ICOSL monoclonal antibody	Phase II trial (NCT02334306)
MHV370	A dual antagonist of human TLR7 and TLR8 that suppresses cytokine production and interferon‐stimulated gene expression	Clinical studies in healthy adult volunteers
RSLV‐132	An RNase Fc fusion protein, target circulating immune‐stimulating RNA associated with immune complexes to inhibit the production of IFN	In Phase II trials (NCT03247686)

### 6.1 Systemic Therapy

Systemic disease serves as a critical prognostic factor in SS, being linked to organ dysfunction mediated by autoimmune processes, which may ultimately become irreversible. Patients with active systemic disease should only receive systemic treatments that modulate or suppress the immune system, including GCs, antimalarials, immunosuppressive drugs, intravenous immunoglobulins, and biologics. This should happen only after a careful assessment of each organ's severity and damage, as not every patients with active systemic disease necessitate systemic treatment [93]. Generally, the management of systemic manifestations of SS should adhere to a two‐phase strategy. The initial phase involves an intensive immunosuppressive strategy to quickly restore organ function (induction of remission), and this is succeeded by a phase focused on sustaining the initial therapeutic response (maintenance of remission). Regrettably, there is a lack of available data on SS patients to substantiate specific recommendations regarding the necessity or duration of induction and maintenance therapy; thus, treatment decisions should be individualized.

### 6.2 Therapies of JAK Activation and Interferon Production

**FIGURE 5:**
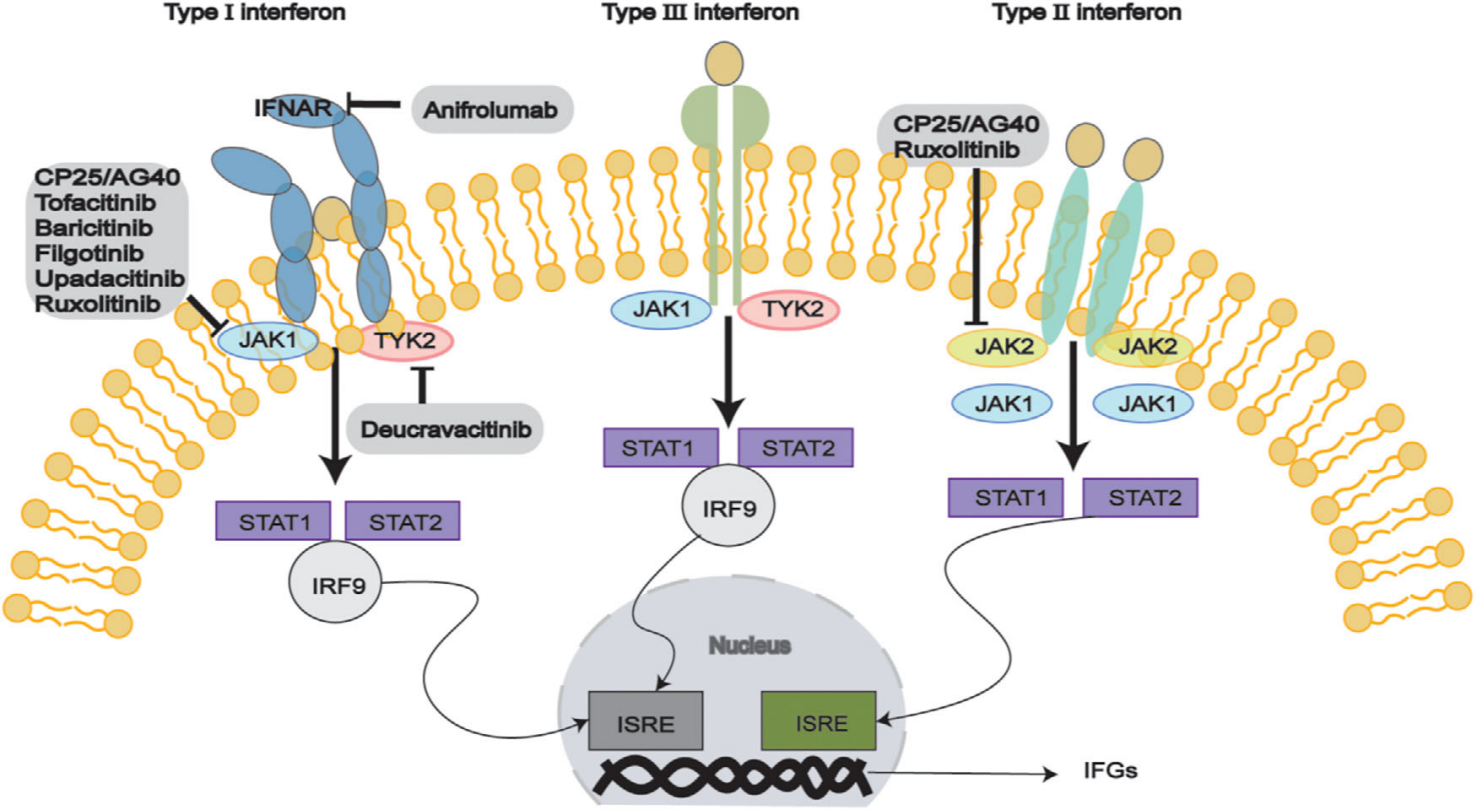
Type I, type II, and type III interferon signaling and IFN‐I pathway‐related inhibitors. Type I, type III, and type II interferons activate different typical signaling pathways leading to downstream induction of interferon‐sensitive response element (ISRE)‐driven and IFN‐γ‐activation site (GAS)‐driven target genes. IFN‐I binds the IFNAR receptor complex (composed of IFNAR1 and IFNAR2); type II interferon binds the IFNGR receptor complex (IFNGR1 and IFNGR2); and type III interferon binding IFNLR receptor complex (composed of INFLR1 and IL‐10Rβ). Binding of interferons to specific receptors results in activation of the Janus kinase (JAK)‐signal transducer and activator of transcription (STAT) pathway. Transcriptional activation of the IFN‐I signaling pathway requires that STAT dimers bind interferon regulatory factors (IRFs), that is, IRF9, translocated into the nucleus, where they bind to interferon signaling genes (IFGs) promoters on the ISREs or GASs, and binding to these promoter elements leads to the transcription of hundreds of IFGs.

The essential role of the JAK‐STAT pathway has led to a rising interest in employing small molecules to prevent JAK activation and the subsequent generation of interferons (Figure 5). Anifrolumab is a monoclonal antibody that targets the IFN‐I receptor (IFNAR) and is presently undergoing Phase II clinical trials (NCT05383677). Filgotinib, a JAK inhibitor, enhanced the salivary flow rate and reduced lymphocyte infiltration in the SGs of mice. A positive correlation exists between IFN‐I‐induced gene signatures and anti‐Ro/SSA and anti‐La/SSB autoantibodies, ESSDAI scores, inflammatory biomarkers, and BAFF mRNA levels in pSS patients. Notably, IFN‐K demonstrates therapeutic potential by modulating immune responses—it reduces IFN‐I activity and ameliorates disease manifestations in murine models while offering advantages over monoclonal antibodies [200], including pan‐IFN‐α neutralization, minimal immunogenicity, cost‐effectiveness, and favorable safety profile. Clinical evidence supports IFN‐α efficacy and safety when administered orally for enhancing salivary secretion in pSS patients, positioning IFN‐K as a promising therapeutic candidate warranting further clinical development [201, 202]. A randomized, Phase II (NCT03100942) in 2022 [203] demonstrated that filgotinib treatment reduced IFN‐I signature activity in patients with SS. While there were no notable differences between the filgotinib and placebo groups for the main or secondary outcome, patients with a baseline ESSDAI score of 14 or higher seemed to show more significant improvements from baseline [204]. The findings of this study could be useful for future research that seeks to evaluate drug tolerance and carry out subgroup analyses.

Clinical trials in Phases I and II for other JAK inhibitors, tofacitinib (NCT05087589/NCT04496960) and baricitinib (NCT04916756/NCT05016297) are currently underway. Tofacitinib, which selectively inhibits JAK1 and/or JAK3, is believed to alleviate lung symptoms of pSS‐related ILD due to its anti‐inflammatory and antifibrotic effects. The clinical trial (NCT 04496960) is designed to assess the effectiveness and safety of oral tofacitinib versus traditional therapy using cyclophosphamide (CYC) combined with azathioprine (AZA) for treating pSS‐ILD [205]. Upadacitinib is a selective JAK‐1 inhibitor specifically indicated for the clinical management of Crohn's disease or RA. Upadacitinib has been clinically observed to have potential therapeutic effects in SS, with significant anti‐inflammatory and antiapoptotic effects in SS [206]. Additionally, Paeniflorin‐6′‐O‐benzene sulfonate (CP25) has been found to inhibit CXCL13 signaling through the JAK‐STAT1/2 pathway [207]. In a mouse model of SS, JAK inhibitors significantly increased salivary flow rate, as well as decreased SG lymphocyte infiltration and IFN‐I features. Promising outcomes have also been observed in animal models of SS using JAK1/2 inhibitors AG490 and ruxolitinib, along with the TBK1 inhibitor BX795 [208]. However, more research is needed to explore JAK inhibitors' role in SS.

The study investigated the association between RNA or protein IFN‐I signatures and the effectiveness of leflunomide‐hydroxychloroquine treatment. The team analyzed data from a double‐blind, randomized, placebo‐controlled clinical trial evaluating leflunomide‐hydroxychloroquine combination in SS patients [209]. The study found that 24‐week changes in ESSDAI scores were exclusively associated with variations in three interferon‐induced proteins: interferon‐induced GTP‐binding protein Mx1, galectin‐9, and CXCL10. No association was observed with alteration in IFN‐I RNA signatures or serum IgG antibody titers [210]. The NECESSITY project has initiated a new multicenter clinical trial to evaluate the combination of leflunomide or mycophenolate mofetil with hydroxychloroquine. This research will deliver essential information on how existing biomarkers perform in patient stratification and their utility as predictive markers of treatment response.

### 6.3 Therapies Targeting B Cells

Elevated B lymphocyte stimulating factor (BLyS) and B‐cell hyperactivity are features of pSS. Belimumab and other anti‐BlyS treatment have been found to increase peripheral memory B cells, reduce naïve, activated, and plasma B‐cell subsets, and enhance the selectivity of B‐cell reconstitution [211]. In contrast, anti‐CD20 treatments such as rituximab focus on targeting and reducing circulating B cells with CD20 expression, yet they show limited success in depleting CD20^+^ B cells that are present in tissues. According to Phase II double‐blind trial (NCT02631538) [212], the safety profiles of belimumab and rituximab in pSS were found to be consistent with those observed in monotherapy. Notably, using both belimumab and rituximab together led to a more substantial reduction of B cells in the SGs than using either drug alone, and the association of greater numerical improvements in total ESSDAI score, the percentage of ESSDAI responders, and stimulated salivary flow, potentially leading to enhanced clinical outcomes.

As a humanized IgG monoclonal antibody, epratuzumab targets CD22 to selectively modulate B cells [213]. The effectiveness and safety of epratuzumab in patients with SLE and related SS were evaluated in a 2018 clinical trial [214]. Epratuzumab treatment led to a notable decrease in disease activity, B‐cell counts and IgM levels in these patients. These findings suggest epratuzumab warrants further investigation as a potential therapy for SS. CD40‐CD154‐mediated interactions between T cells and B cells in primary desiccation syndrome contribute to abnormal lymphocyte activation within inflamed tissues, culminating in inflammation of the SGs and damage to other tissues. The safety and preliminary efficacy of iscalimab (CFZ533) [215], a new anti‐CD40 monoclonal antibody, were evaluated in a multicenter, randomized, double‐blind clinical trial (NCT02291029) involving individuals with primary dry syndrome. The ESSDAI scores decreased by an average of 5.21 points with intravenous iscalimab compared to placebo, whereas the subcutaneous group did not show a significant change.

B‐cell dysfunction is a key factor in the development of SS, and artemisinin has now been found to target B cells. Artemisinin, an orally administered derivative, inhibits BAFF‐induced NF‐κB activation, leading to improved saliva flow rates [216]. Another investigational agent that has completed Phase I trials (NCT02614716) is tibulizumab, a bispecific dual antagonist antibody targeting both BAFF and IL‐17A. JH013 is an antibody of human origin that aimed at the BAFF receptor. It selectively binds to the BAFF receptor on B cells, inducing cell lysis and blocking BAFF‐mediated signaling. This mechanism offers a new treatment strategy for autoimmune diseases characterized by B‐cell hyperactivation, such as pSS and SLE. Currently, a Phase I clinical trial is underway to evaluate the safety, tolerability, pharmacokinetics, and pharmacodynamics of JH013 injection in pSS patients. Telitacicept is a novel, fully human TACI‐Fc fusion protein that attach to both BAFF and APRIL, cytokines critical for B‐cell differentiation, maturation, and antibody secretion by plasma cells [217]. Overexpression of BAFF/APRIL has been linked to the development of several autoimmune disorders, such as RA, SLE, and IgA nephropathy [218]. In a Phase II randomized, double‐blind, placebo‐controlled trial (NCT04078386), the effectiveness and safety of telitacicept were evaluated in pSS patients [219]. Telitacicept (160 mg) treatment significantly reduced in ESSDAI scores from baseline to Week 24 compared to the placebo group, along with marked decreases in MFI‐20 (Multidimensional Fatigue Inventory) and serum immunoglobulin levels. Currently, a Phase III clinical trial is underway to further assess telitacicept in pSS patients.

The cytokine BAFF, induced by IFN, is crucial for B‐cell activation, survival, and the lifespan of plasma cells. In recent Phase IIb trials (NCT02962895), the BAFF receptor inhibitor ianalumab (VAY736) and the NF‐κB activity inhibitor iguratimod have shown a potential to improve disease activity [1, 220]. A human monoclonal antibody, ianalumab (VAY736), works by killing B cells in two main ways—by directly killing the cells or by blocking the BAFF receptor and further interfering with the signaling pathway [1]. A randomized, double‐blind, two‐arm, extension Phase III study is currently underway to assess the safety and effectiveness over an extended period of ianalumab (VAY736) in SS patients following the NEPTUNUS trial. Deucravacitinib, taken orally, selectively inhibits TYK2 allosterically, impacting the signaling of IFN‐I, IL‐23, and IL‐12 [221]. Published results from a Phase II clinical trial show the effects of deucravacitinib on patients with moderate to severe SLE [222], demonstrating that the drug inhibits the IFN‐I and B‐cell pathways, offering a molecular basis for its potential therapeutic role in desiccation syndrome. A Phase III, randomized, double‐blind, placebo‐controlled study to assess the safety and effectiveness of two therapeutic doses of deucravacitinib for treating patients with active dry syndrome is ongoing. These findings establish a molecular basis for comprehending its potential therapeutic application in SS.

**FIGURE 6:**
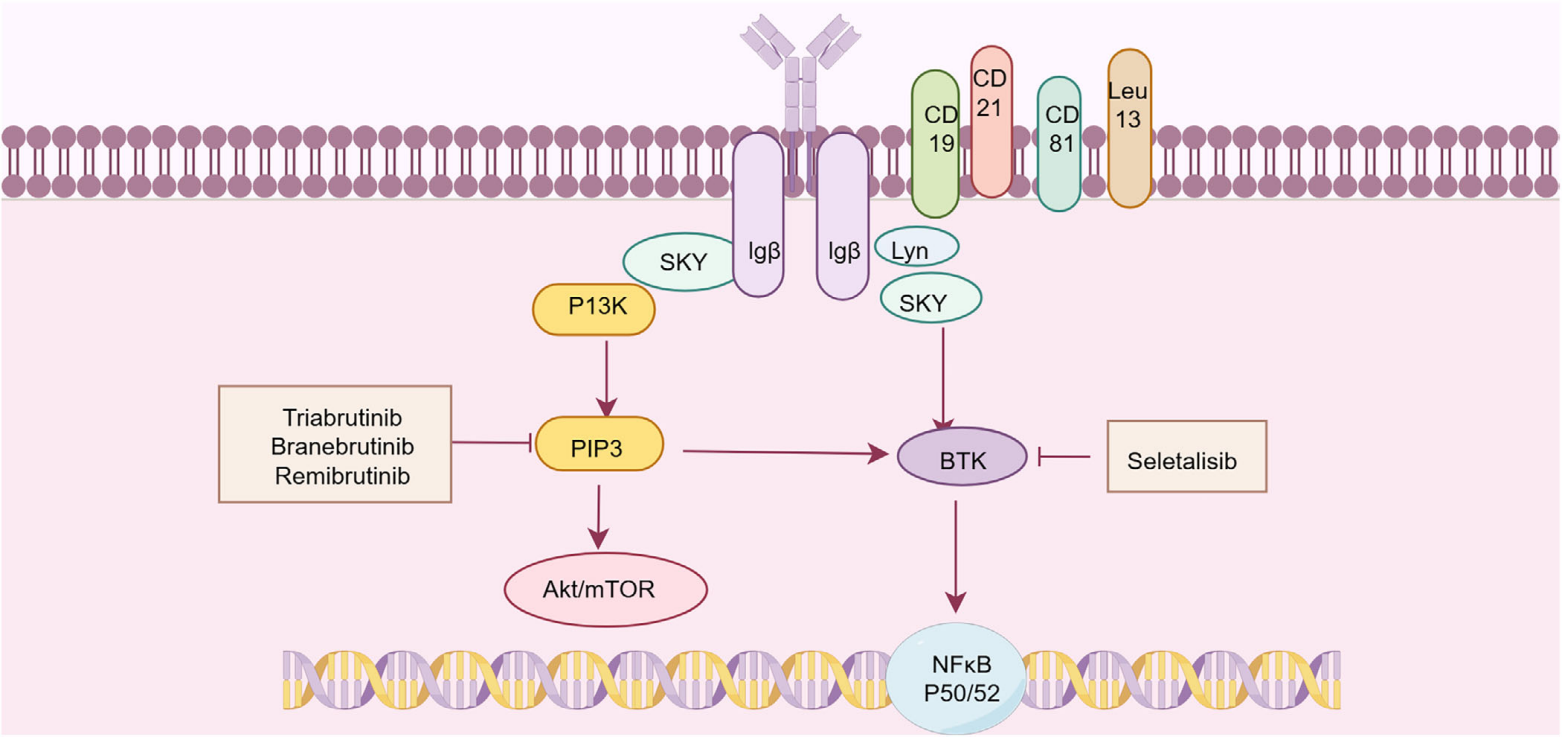
Biological and synthetic DMARDs that target BCR signaling. Targeting BCR signaling has emerged as a promising therapeutic strategy in SS, the BCR signaling cascade is remarkably complex, involving a ged as a promising therapeutic strategy in SS, the BCR signaling cascade is remarkably complex, inK signaling enzymes, ultimately driving diverse cellular outcomes ranging from survival, anergy, or apoptosis to proliferation and differentiation into antibody‐producing cells or memory B cells. Created with www.figdraw.com.

Targeting BCR signaling has emerged as a promising therapeutic strategy in SS, complementing existing approaches focused on B‐cell depletion or surface molecule targeting. The BCR signaling cascade is remarkably complex, consisting of a “signalosome” that includes the BCR, tyrosine kinases, and PI3K signaling enzymes, ultimately driving diverse cellular outcomes ranging from survival, anergy, apoptosis, proliferation, and differentiation into antibody‐producing or memory B cells (Figure 6) [223]. Among BTK‐targeted therapies, a clinical trial of tirabrutinib failed to demonstrate significant differences between treatment and placebo groups for both primary and secondary endpoints [203]. However, branebrutinib is undergoing evaluation in a Phase III study (NCT04186871) for SS, while remibrutinib has shown preliminary efficacy with a favorable safety profile in Phase II studies [224]. Among PI3K inhibitors, clinical evaluation of seletalisib has demonstrated a trend toward clinical improvement [225]. Histopathological analyses revealed encouraging effects of seletalisib on SG inflammation and tissue architecture. Parsaclisib exhibited therapeutic efficacy in two spontaneous SS murine models, ameliorating SG inflammatory severity while reducing circulating autoantibody titers and salivary BAFF (BLyS/BAFF) concentrations [226]. Collectively, even with some contradictory findings, focusing on B‐cell signaling pathways represents a promising therapeutic approach for SS. Ongoing and future clinical trials will further elucidate the potential efficacy of this strategic intervention.

A promising therapeutic approach involves targeting the neonatal Fc receptor (FcRn) to reduce circulating IgG antibodies, including harmful autoantibodies. Nipocalimab, a human monoclonal antibody with strong affinity, employs this FcRn‐targeted approach to effectively reduce circulating IgG levels [227]. Mechanistically, blocking FcRn specifically inhibits the interaction between natural IgG and FcRn, thereby redirecting these antibodies toward lysosomal degradation and subsequent depletion [228]. This targeted enhancement of endogenous IgG degradation may constitute an effective treatment approach for SS patients. Currently, a Phase II clinical trial (NCT04968912) is evaluating nipocalimab in SS, while investigations are ongoing for another FcRn antagonist, efgartigimod alfa (NCT05817669).

### 6.4 Therapy of T Cells

Various methods of targeting T cells in desiccation syndrome are being investigated. To examine the effectiveness, safety, and immune response of low‐dose IL2 (LD‐IL‐2) in treating pSS through a double‐blind, placebo‐controlled, randomized clinical trial (NCT02464319) [229]. LD‐IL‐2 was shown to be effective and well tolerated in patients with pSS in this randomized clinical trial, achieving immune homeostasis restoration by increasing regulatory T cells and CD24^hi^ CD27^+^ B cells. The human monoclonal antibody ustekinumab focuses on the shared p40 subunit common to both IL‐12 and IL‐23, has demonstrated efficacy in ameliorating articular manifestations in SS patients according to case reports [230]. Preliminary clinical investigations evaluating ustekinumab for SS are currently underway (NCT04093531). The B‐ and T‐lymphocyte attenuator (BTLA) functions as an inhibitor of T‐cell activation and proliferation. Mechanistically, both TCR‐mediated proliferation and cytokine production in T cells are suppressed by BTLA, which also curtails B‐cell proliferation [231]. A Phase II clinical trial is assessing the therapeutic potential of venanprubart (a BTLA‐targeted agent) in SS patients [232].

Costimulation is undeniably a key factor in the continuation and intensification of the autoimmune response to SS. IBI355 is a CD40L antibody. CD40L antibodies are used to control immune cells activation and function by blocking the binding of CD40L to CD40, which results in the suppression of abnormal immune cell activation and a decrease in inflammatory responses. A safety, tolerability, and pharmacokinetic study evaluating multiple dosing of IBI355 in pSS subjects is underway in a Phase I trial. Isocalimab (CFZ533), a fully human IgG1 anti‐CD40 monoclonal antibody, demonstrated significant improvement in ESSDAI scores with intravenous administration in Phase II clinical trials (NCT04541589). Dazodalibep, an innovative TN3 fusion protein serving as a CD40L antagonist, not involving antibodies, showed comparable efficacy [233]. Notably, dazodalibep treatment resulted in clinically meaningful ESSDAI improvements compared to placebo in two separate groups of patients: those experiencing moderate‐to‐high levels of systemic disease activity, and those with a significant symptom burden but minimal systemic involvement (characterized by ESSPRI ≥ 5 with ESSDAI < 5) [233]. Building upon these promising outcomes, the TWINSS core trial's extension study is currently examining isocalimab in more diverse SS populations [234], while two Phase III clinical trials (NCT06104124/NCT06245408) are being planned to further assess dazodalibep's therapeutic potential.

Abatacept, a cytotoxic T‐lymphocyte protein 4 (CTLA‐4)‐immunoglobulin fusion protein, represents the first pharmacological agent developed to modulate T‐cell costimulation in SS by selectively obstruct CD28 interactions with CD80/CD86 [235]. During the open‐label extension phase of the ASAP‐III trial, abatacept treatment demonstrated sustained improvements across multiple outcome measures after 48 weeks, including clinical assessments, patient feedback, dry eye symptoms, and laboratory data [236]. The ICOS–ICOSL axis represents another biologically significant pathway involved in germinal center formation. ICOSL is abnormally expressed on B cells, APCs, and SGECs in SS, facilitating T‐cell‐dependent activation of B cells [237]. In a Phase II randomized controlled trial (NCT02334306), prezalumab, an anti‐ICOSL humanized monoclonal antibody, was assessed for SS. However, prezalumab treatment did not consistently improve clinical endpoints or biomarker profiles of disease activity.

Artemisinin ameliorates pSS by restoring Treg/Th17 cell balance. KEGG pathway analysis revealed that artemisinin's therapeutic effects involve the IL‐17, HIF‐1, apoptosis, Th17 differentiation, PI3K‐Akt, and MAPK signaling pathways. Molecular docking demonstrated a high binding affinity between artemisinin and key nodes of the IL‐17 signaling. Studies in vivo showed that artemisinin brought back the functionality of SGs, alleviated glandular damage, increased Tregs, and suppressed IL‐17 secretion in NOD/Ltj mice [238]. Aberrant recognition of RNA‐containing autoantigens by TLR7 and TLR8 contributes to the development of diseases. MHV370 is a dual antagonist for human TLR7 and TLR8 that suppresses cytokine production and ISG expression in both in vitro and in vivo contexts, and has demonstrated effectiveness in murine lupus models [239]. Clinical studies in healthy adult volunteers were conducted to examine the safety, tolerability, pharmacokinetics, and pharmacodynamics of MHV370 when administered in single and multiple doses [240]. The data support the continued development of MHV370 for systemic autoimmune diseases associated with heightened TLR7 and TLR8 activation. Hydroxychloroquine, a TLR inhibitor, as a result of modulating endosomal pH and inhibiting TLR‐7 and 9 receptor activation by endogenous nucleic acids, hydroxychloroquine influences IFN‐I production by pDCs in lupus patients [241, 242, 243]. While hydroxychloroquine demonstrated efficacy in reducing IFN‐I characteristics, improving skin manifestations, and acting as an alternative to steroids in a clinical study, it did not significantly improve symptoms of SS [244, 245].

RSLV‐132, a novel drug incorporating RNase within the IgG1 Fc domain, aims to target circulating immune‐stimulating RNA associated with immune complexes to inhibit the production of IFN [232]. RSLV‐132 was investigated further in a Phase II randomized, double‐blind, placebo‐controlled trial for pSS patients (ClinicalTrials.gov identifier: NCT03247686). The study enrolled 28 pSS patients who displayed an IFN‐I response and upregulation of anti‐Ro/SSA antibodies. Results indicated that an upregulation of selective interferon‐inducing genes following treatment with RSLV‐132 was associated with a reduction in fatigue. This study posits that pharmacological interventions, specifically nuclease therapy with RSLV‐132, may ameliorate the severe fatigue commonly observed in patients with pSS, despite the intricate and derivative nature of the biomarker data [232, 246]. The recent Phase II clinical trial of RSLV‐132 [247], assessing ribonuclease in patients with severe fatigued following SARS‐CoV‐2 infection, yielded intriguing results [248].

### 6.5 Chimeric Antigen Receptor‐Modified T Cell Treatment

In recent years, genetically modified chimeric antigen receptor (CAR) T cells have become a hopeful immunotherapy strategy for a range of diseases. CAR T cells are T lymphocytes that have been genetically altered to display a surface receptor called CAR, designed to target specific homologous antigens on cells [249]. The demonstrated efficacy of CAR T‐cell therapy in treating hematologic malignancies (leukemia, myeloma, and NHL) has prompted consideration of its potential application for autoimmune diseases. Compared to antibody‐based therapies, CAR T cells exhibit faster onset and more durable effects. Engineered to target CD19 and other B‐cell surface antigens, CAR T cells have achieved remarkable success in treating relapsed or refractory B‐cell malignancies [250]. A systematic review analyzed 80 autoimmune disease patients treated with CAR‐T cell therapy, including 52 with SLE, 16 with SSc, seven with idiopathic inflammatory myopathy, two with antiphospholipid antibody syndrome, two with RA, and one with SS. Among these patients, 44 received CD19‐targeted CAR‐T therapy while 36 were treated with combined BCMA/CD19‐targeted CAR‐T therapy. Remarkably, all patients achieved an immunosuppression‐free status at final follow‐up, with no fatal adverse events reported [251].

Although the application of CAR‐T therapy in SS remains exploratory, preliminary data from other autoimmune indications demonstrate therapeutic potential with an acceptable safety profile [252, 253]. Currently, a Phase I/II clinical trial evaluating bispecific CD19 and BCMA‐directed CAR‐T is being conducted in patients with treatment‐refractory SS (NCT05085431). These findings demonstrate that CAR‐T cell therapy is safe and effective for refractory autoimmune diseases. However, future studies are imperative to validate these results, investigate long‐term outcomes, and optimize treatment protocols to enhance both efficacy and safety. This innovative therapeutic approach holds promise for achieving durable remission in autoimmune disorders and could provide substantial clinical benefits for affected patients. Current research focuses on targeting various surface markers including CD19, CD20, and BCMA [254]. Notwithstanding these promising results, CAR‐T therapy faces several challenges: time‐consuming cell manufacturing processes, complex production protocols, high treatment costs, and potential severe adverse effects that may complicate clinical management.

### 6.6 Pharmacology in the Treatment of SS

SS is an autoimmune disorder that can result in gastrointestinal complications, including constipation and intestinal inflammation. *Paeonia lactiflora* Pall, as a medicinal substance, possesses a wide range of pharmacological properties and is a crucial component in numerous pharmaceutical formulations [255]. Total glucoside of *Paeonia lactiflora* (TGP), composed of bioactive compounds from its root, has shown efficacy in reducing inflammation and decelerating disease progression in experimental autoimmune diseases models, such as RA, SLE, desiccation syndrome, and psoriasis [256]. TGP positively influences various immune cells by modulating the activation, proliferation, differentiation, and effector molecules production in T cells, macrophages, and dendritic cells [257]. It exhibits extensive immunomodulatory impacts on different immune cells. TGP influences intracellular signaling pathways, such as JAK/STAT, NF‐κB, MAPK, and PI3K/Akt/mTOR. Furthermore, TGP has been effectively used in managing autoimmune diseases, demonstrating satisfactory results with minimal side effects [258].

Oxidative stress can lead to necrosis and apoptosis of lacrimal gland cells, resulting in impaired secretory function or decreased tear production. *Moringa oleifera* leaf extract has strong anti‐inflammatory and antioxidant activities [259]. Research on the impact of *Moringa oleifera* leaf extract affects the histopathology and secretory of the lacrimal gland in SS mice showed that *Moringa oleifera* leaf extract significantly improves the histopathology of the lacrimal gland in SS mice, but was not effectively in increasing tear production [260]. Fangchinoline is a bis benzylisoquinoline alkaloid extracted from powdered anthemis, which is commonly used as an analgesic, antirheumatic, and antihypertensive agent. Recent studies have shown that fangchinoline significantly improves salivary secretion, reduces the number of lymphocytic foci in the submandibular gland, effectively inhibits the proliferation of tumor B lymphocytes, and reduces SS‐like responses in NOD/Ltj mice. This further emphasizes the possible clinical use of antipyrine in treating SS [261]. Furthermore, runzaoling has been demonstrated to downregulate the expression of STAT3 and IL‐17 in the submandibular gland and Th17 cells of NOD mice by modulating the PI3K/Akt/mTOR signaling pathway [262]. This regulation leads to a decrease in the activity of CD4^+^ T lymphocyte differentiation into Th17 cells. Rutin significantly enhances salivary secretion and the SG index in the submandibular gland while inhibiting oxidative stress and inflammatory cytokines. Additionally, rutin has been shown to further ameliorate SS by modulating the CaR/NLRP3/ NF‐κB signaling pathway [263]. Notably, the overexpression of the calcium‐sensitive receptor (over‐CaR) was found to inhibit some of rutin's activity, indicating that CaR could be an essential target for SS treatment [263].

### 6.7 Nonpharmacological Treatment

Considering the intricate nature of the disease and the unsatisfactory results from drug treatments, nondrug strategies might be especially beneficial for addressing fatigue, which is one of the most difficult symptoms of this condition. As of now, no medication tested in multiple clinical trials has led to a significant improvement in fatigue symptoms. Resistance training (RT) has shown promising potential for fatigue reduction with good tolerability. The research by Dardin et al. shown that a structured RT program was effective in improving fatigue in pSS patients [264]. The program exhibited excellent tolerability, good compliance, and no serious adverse effects in this patient population. In patients with SS experiencing mild dysfunction of the glands, nonpharmacological glandular stimulation is recommended as the preferred first‐line treatment with taste boosters including sugar‐free sour candies, lozenges, and xylitol, in addition to mechanical stimulants like sugar‐free chewing gum [93]. All nonpharmacologic interventions provided some relief of subjective symptoms, but this could not be quantified, and there was no compelling proof that one intervention was more effective than the others.

## 7 Conclusion and Prospects

SS is a multifaceted condition with diverse epidemiological and demographic presentations. Despite a seemingly consistent prevalence over time, geographical disparities may impact the population characteristics, disease phenotype, and disease activity levels. The underlying factors driving this heterogeneity, whether environmental or genetic, remain uncertain as they are not mutually exclusive. The current body of evidence regarding the efficacy of therapies specifically targeting SS remains inconclusive, as numerous clinical trials have not met their primary endpoints. Nevertheless, recent investigations focusing on B cells, B–T cell interactions, IFN signaling, and pharmacological interventions have shown encouraging outcomes.

In recent years, there have been major breakthroughs in understanding the characteristics linked to the various clinical and immunological presentations of the disease, however, further research is imperative. A multidisciplinary and customized approach, based on the type and severity of organ involvement, is essential for managing patients with desiccation syndrome.

In this review, which follows other articles in this series, we will synthesize the latest literature on the epidemiology, classification criteria, molecular pathogenesis, diagnosis, and clinical features of SS treatment. This synthesis aims to provide a comprehensive perspective on understanding SS, thereby establishing a critical scientific foundation for future research endeavors and therapeutic strategies.

## Author Contributions

Ying Hu, Bin Wen: Organize literature and original draft writing, search literature, and writing editing; Yukai Jing, Huifeng Shang: Conception and writing review; Yunfei Zhang, Xiaocui Wang, Xuemei Duan, Haonan Li, Yufeng Fan: Writing and revising of the manuscript. All the authors contributed to the writing and revising of the manuscript.

## Ethics Statement

Not applicable.

## Conflicts of Interest

The authors declare no conflicts of interest.

## Data Availability

The authors have nothing to report.
